# Optimization of an Intermittent Finger Endurance Test for Climbers Regarding Gender and Deviation in Force and Pulling Time

**DOI:** 10.3389/fspor.2022.902521

**Published:** 2022-05-23

**Authors:** Claudia Augste, Marvin Winkler, Stefan Künzell

**Affiliations:** Faculty of Philosophy and Social Sciences, Institute for Sport Science, University of Augsburg, Augsburg, Germany

**Keywords:** climbing, rock climbing, lead climbing, testing, fingerboard

## Abstract

Performance diagnostics of finger strength is very relevant in climbing. The aim of our study was to find modalities for an intermittent finger flexor muscle endurance test that optimize the correlation of test performance with lead climbing performance. Twenty-seven female and 25 male climbers pulled with 60% MVC and a work-to-rest ratio of 7:2 s on a fingerboard until fatigue. The highest correlations, *R* = 0.429, were found for women when 9% deviation in the required force and 1 s deviation in the required pulling time was tolerated. For men, the optimum was reached with the same time deviation and a force deviation of 6%, *R* = 0.691. Together with maximum finger strength the repetitions explained 31.5% of the variance of climbing ability in women and 46.3% in men. Consequences from our results are to tolerate at least 7% force deviation for women and 5% for men and to terminate the finger endurance test quickly after the force falls below the threshold.

## Introduction

Scientific monitoring of the new Olympic sport of climbing is increasing. One focus is on performance diagnostics of maximum finger strength and finger endurance, which are very relevant in climbing (MacLeod et al., 2007; Baláš et al., 2012; Philippe et al., 2012; Saul et al., 2019). The test protocols used by individual research groups are quite different (Stien et al., 2022). Two main variations can be identified: In one variation, participants have to perform sustained isometric contractions (Table 1). In the other variation intermittent contractions are used, which prescribe specific rhythms of work and rest (Table 2). Here, the contraction-relax ratios are based on the demands of lead climbing (Michailov, 2014). The performance parameters recorded in previous studies usually refer to the time to failure (TTF), the force-time integral (FTI) or the number of repetitions completed (REP) (Tables 1, 2). For both, continuous and intermittent protocols, different test devices have been used: Hand dynamometers, specially designed measuring apparatuses and the currently most common rungs or holds.

**Table 1:** Overview of measurement methods of finger flexor muscle endurance based on sustained isometric contractions.

**Study**	**Parameter**	**Load**	**Device**
Cutts and Bollen (1993)	FTI	80% MVC	Hand dynamometer
Ferguson and Brown (1997)	TTF	40% MVC	Hand-grip ergometer
MacLeod et al. (2007)	TTF, FTI	40% MVC	Special apparatus
Limonta et al. (2008)	TTF	80% MVC	Hand-grip ergometer
Philippe et al. (2012)	TTF, FTI	40% MVC	Special apparatus
Baláš et al. (2012)	TTF	Finger hang	25 mm ledge
López-Rivera and González-Badillo (2012)	TTF	Finger hang	11 mm ledge
Fryer et al. (2015c)	(TTF,) FTI	40% MVC	Special apparatus
Fryer et al. (2015a)	FTI	40% MVC	Special apparatus
Medernach et al. (2015)	TTF	Finger hang	3 Different grips
Baláš et al. (2016)	TTF, FTI	60% MVC	Special apparatus
Ozimek et al. (2016)	TTF	50% MVC	Hand dynamometer
		Finger hang	25 mm ledge
			40 mm ledge
Ozimek et al. (2017)	TTF	Finger hang	25 mm ledge
			40 mm ledge
Hermans et al. (2017)	TTF	Finger hang	25 mm ledge
Bergua et al. (2018)	TTF	Finger hang	14 mm edge
	Minimum edge depth	40's-Finger-hang	Edge
Michailov et al. (2018)	F_avg_, I_fatigue_	30's All out test	Special apparatus
López-Rivera and González-Badillo (2019)	TTF	Finger hang	11 mm edge
Baláš et al. (2021)	TTF, FTI	60% MVC	23 mm ledge
Rokowski et al. (2021)	TTZ, FTI, FTI/kg	60% ± 10% MVC	23 mm ledge

*TTF, time till task failure (s); FTI, force-time integral (Ns); F_avg_, average force_;_ I_fatigue_, fatigue index; TTZ, time in target zone (s)*.

**Table 2:** Overview of measurement methods of finger flexor muscle endurance using intermittent isometric contractions.

**Study**	**Parameter**	**Load**	**Work-to-rest ratio**	**Device**
Ferguson and Brown (1997)	TTF	40% MVC	5:2 s	Hand-grip ergometer
Vigouroux and Quaine (2006)	REP	80% MVC	5:2 s	Special apparatus
MacLeod et al. (2007)	TTF FTI	40% MVC	10:3 s	Special apparatus
Philippe et al. (2012)	TTF FTI	40% MVC	10:3 s	Special apparatus
Fryer et al. (2015a)	FTI	40% MVC	10:3 s	Special apparatus
Fryer et al. (2015b)	FTI	40% MVC	10:3 s	Fingerboard apparatus
Medernach et al. (2015)	TTF	Hang	8:4 s	Fingerboard
Baláš et al. (2016)	TTF, FTI	60% MVC	8:2 s	Special apparatus
Michailov et al. (2018)	REP, TTF, FTI	60% MVC	8:2 s	Special apparatus
Giles et al. (2019)	TTF	80, 60, 45% MVC	7:3 s	20 mm rung
Stien et al. (2019)	TTF	60% MVC	7:3 s	23 mm ledge
Baláš et al. (2021)	TTF, FTI	60% MVC	8:2 s	23 mm ledge
Giles et al. (2021)	End-test force	100% MVC	7:3 s	20 mm rung
Rokowski et al. (2021)	TTZ, FTI, FTI/kg	60 ± 10% MVC	8:2 s	23 mm ledge

*TTF, time till task failure (s); FTI, force-time integral (Ns); REP, repetitions till task failure (#)*.

Many studies have used the above presented parameters to examine performance differences between climbers and non-climbers (MacLeod et al., 2007; Philippe et al., 2012), between climbers of different ability levels (Fryer et al., 2015a,c; Ozimek et al., 2017; Bergua et al., 2018), or between different climbing disciplines (Michailov et al., 2018; Stien et al., 2019). However, some studies also focus on how well measured finger endurance performance predicts climbing performance. Two points are striking here. First, regarding the relationship of endurance tests to climbing performance, there have been some studies to date on sustained contractions (López-Rivera and González-Badillo, 2012; Ozimek et al., 2016; Bergua et al., 2018; Michailov et al., 2018; Baláš et al., 2021). For intermittent tests, however, the evidence is still relatively limited. To our knowledge, only two studies consider the relationship of intermittent testing to climbing performance (Baláš et al., 2021; Rokowski et al., 2021).

Second, a closer look reveals that the termination criteria for test execution are selected quite differently. In Baláš et al. (2021), for example, the test is automatically terminated by the software if a threshold value is undershot. In other studies, the test is stopped by the participant due to volitional fatigue (MacLeod et al., 2007; Fryer et al., 2015a). Also, this threshold value varies from study to study from 5% (Limonta et al., 2008; Philippe et al., 2012; Fryer et al., 2015b) to 10% (Baláš et al., 2021), or one standard deviation (Giles et al., 2021), respectively. Further variations are found for the duration for which the value falls below the threshold. For example, some tests are terminated with a tolerance of 1 second (Baláš et al., 2021), in other studies a tolerance of 2 s is allowed (Philippe et al., 2012; Fryer et al., 2015c). It is a bit of a dilemma to decide when the repetition is not valid anymore. Because when worsening fatigue is being experienced, the value continues to fall slowly.

It seems reasonable to us to look for implementation modalities for frequently used tests that increase the validity of the tests. Therefore, the main goal of our study was to find out which test termination criteria in intermittent finger flexor muscle endurance tests optimize the correlation of test performance with climbing performance. Furthermore, we wanted to find out how well the optimized endurance test together with the athletes' maximum strength predicts lead climbing performance.

## Materials and Methods

### Participants

Calls for study participation were made via social media as well as announcements in local climbing gyms. The precondition was that participants had to be at least 16 years old and climb regularly, no matter at what climbing grade. All individuals who responded within the allotted period and met the criteria were invited for the study. Further consideration was given to all those who could indicate the three highest grades of lead climbing difficulty they had climbed in redpoint mode within the last 12 months. According to Draper et al. (2011) the average of these difficulty levels represented the dependent variable climbing performance in the test and was reported on the IRCRA scale (Draper et al., 2015). Thus, a total of 52 climbers (27 female, 25 male) finally participated in the study (Table 3). They were on average 29.1 ± 6.6 years old and had 5.6 ± 5.2 years of climbing experience. Their climbing skill ranged from 11 to 24 on the IRCRA scale. According to the IRCRA scale classification (Draper et al., 2015), 34 athletes were intermediate, 17 advanced and one elite. Seven were left-handed and 45 were right-handed.

**Table 3:** Participant characteristics.

	**Women**	**Men**	**Total**
N	27	25	52
Age [years]	29.4 ± 7.6	28.8 ± 5.4	29.1 ± 6.6
Climbing experience [years]	5.8 ± 3.9	5.3 ± 5.0	5.6 ± 3.6
Lead climbing skill [IRCRA]	14.2 ± 2.3	16.1 ± 2.8	15.1 ± 2.7
Climbing classification	15 intermediate 12 advanced	19 intermediate 5 advanced 1 elite	34 intermediate 17 advanced 1 elite
Handedness	5 left, 22 right	2 left, 23 right	7 left, 45 right
Absolute maximum force dominant hand [kg]	44.0 ± 8.2	62.4 ± 7.1	52.8 ± 12.0
Absolute maximum force non-dominant hand [kg]	42.6 ± 8.9	60.0 ± 8.2	50.1 ± 12.2
Relative maximum force dominant hand [% body weight]	70.8 ± 12.7	85.5 ± 11.2	77.5 ± 14.0
Relative maximum force non-dominant hand [% body weight]	68.3 ± 13.1	82.3 ± 13.6	74.7 ± 15.0

The experiments were undertaken with the understanding and written consent of each participant, and the study conforms with Declaration of Helsinki. The Ethics Committee of the Augsburg University, Germany, approved our research (approval number 20/104, 03 April 21).

### Procedure

In a cross-sectional study, we conducted a maximum finger flexor muscle strength test and an endurance test on a fingerboard.

The participants started with a standardized 15-min warm-up consisting of a general warm-up of the involved muscles and a specific warm-up on the fingerboard. They performed 3 series of 5 repetitions of intermittent pulling (7 s pulling, 2 s rest) with increasing load. The first two series were performed two-handed, and the last one one-handed with 50, 70 and 50% of the body weight, respectively. We took the body weight because the maximum force was not known yet. The intermittent pulling within the warm-up served at the same time for familiarization for the following endurance test. Thereafter, 2 preparatory trials for the maximum strength test were performed.

For the maximum strength test, the participants stood on an Entralpi© force plate and had to pull alternately with the right and left hand as hard as they could for 5 s on a 23 mm deep rung with 12 mm radius with a half crimp finger position. This procedure was repeated three times. There was a 10-s rest between the right- and left-hand repetitions, and a 3-min rest between each of the three trials. The maximum strength (MVC) of each hand measured with the Entralpi© app was set to the maximum of the 3 repetitions. The results are shown in Table 3. Averaged over the right and left hand, the athletes were able to pull 51.9 ± 11.9 kg, which corresponds to 76.1% ± 14.2% of their body weight.

**Figure 1:**
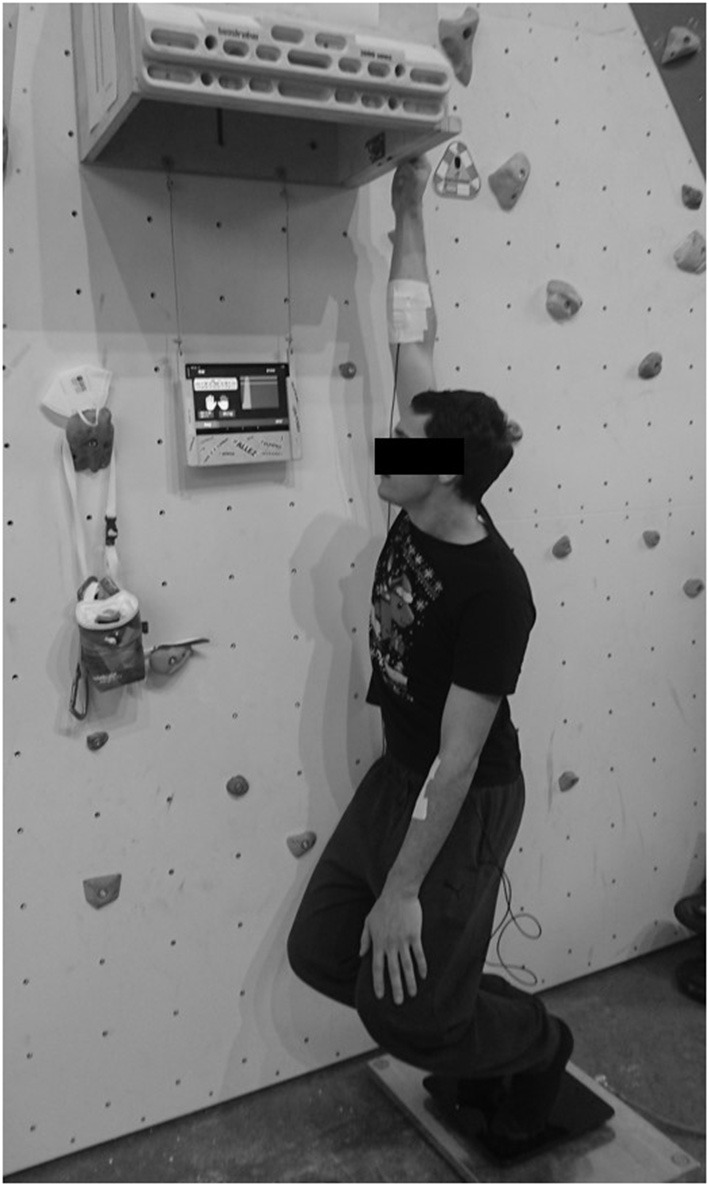
Test setup. Participant standing on Entralpi© force plate, tablet for visual control at head height, one-hand pull on 23-mm rung.

Thereafter, the intermittent endurance test was performed, which should provide the basis to determine the relationship between finger flexor muscle endurance and climbing performance. For this purpose, the participants had to pull on the 23 mm deep rung in a rhythm of 7 s load and 2 s rest with 60% of the previously determined maximum force. The rung size of 23 mm had been chosen because it targets both finger flexor muscles optimally and is therefore used in many studies about finger strength and endurance testing (Baláš et al., 2021; Rokowski et al., 2021; Stien et al., 2021). The load and rest rhythm durations have been chosen because they map the load structure of lead climbing competitions (Winkler et al., 2022). Participants were randomly chosen to begin with the dominant or non-dominant hand. After a 5-min rest, they performed the test with the other hand. The participants were instructed to pull the determined force throughout the 7 s. In the 2-s rest, the athletes could put down and shake the arm and, very quickly, chalk their fingers. A tablet mounted approximately at head height displayed a line for the force value to be targeted (60% MVC) and the force-time curve in real time via the Entralpi© app, allowing participants to monitor whether they still generated the required force (Figure 1). Participants could stop the test at any time if they felt uncomfortable or if they could not manage another repetition. However, this occurred in only about 5% of cases. Normally, the test was terminated by the experimenter as soon as the force exerted by the athlete dropped 10% below the the required force for an extended period longer than 2 s in two consecutive trials.

### Statistical Analysis

After the test had been performed, the recorded data were evaluated retrospectively. To analyze which criterion one should apply in order to obtain the greatest correlation of the test with climbing performance, we calculated bilateral correlations between the lead climbing ability and the number of repetitions for different criteria for a valid repetition. For this purpose, all possible constellations of negative deviation from the required 60% MVC in one-percent increments up to a maximum of 10% undercutting and a deviation from the required pull duration of 7 s in 1-s decrements were considered. When considering the pulling time, only the time within the respective force target zone was taken into account. Also, the time at the beginning of the pulling was not taken into account until the target zone was reached. Lead climbing ability was assessed using the self-reported value on the IRCRA-scale as shown above.

To determine both the proportion of endurance and of maximum strength in lead climbing performance, regression analyses were performed. After adjusting for outliers, the data met the assumptions for regression analysis. All variables had equal variance and were found to be homoscedastic and normally distributed. The inclusion method was used with the best value from the above endurance test analysis for women and men, respectively, and the value of the relative maximum strength. The beta coefficient was consulted as an indicator of the level of influence on climbing performance. Analyses were performed using SPSS software (IBM SPSS, Version 26.0). The level of significance was set at α < 0.05 for each procedure.

## Results

### Number of Repetitions for Different Criteria

Tables 4, 5 show the number of repetitions on the fingerboard for women and men, respectively, for different criteria for a repetition to be counted as a valid repetition. Logically, the number of repetitions is lower when the criteria are stricter. Thus, on the one hand, the number of repetitions is reduced when the period of time over which the force must be applied during the pull is set longer. On the other hand, the number of repetitions is lower if a smaller decrease of force is tolerated. For women, there was little variation in the number of repetitions for the different tolerated decreasaes in pulling time. Considering that the force is mostly built up within the first second at the beginning of the repetition this means that the requirement to pull for 7 s was basically followed quite well by the women. With regard to the tolerated force deviation, it can be seen that the women hardly succeeded in meeting the specified force exactly. If no deviation was tolerated, they achieved just under 5 repetitions; if 10% deviation was tolerated, they achieved almost 10 repetitions.

**Table 4:** Number of repetitions for different criteria of a “valid” repetition in women.

**Tolerated decrease in force**			**Tolerated decrease in pulling time**	
			**0–1 s**	**1–7 s**
0%	REP	M	4.7	4.7
		SD	3.2	3.2
1%	REP	M	5.5	5.6
		SD	3.5	3.5
2%	REP	M	5.9	6.1
		SD	3.4	3.6
3%	REP	M	6.7	6.9
		SD	3.4	3.6
4%	REP	M	7.4	7.5
		SD	3.7	3.9
5%	REP	M	7.8	7.9
		SD	3.6	3.9
6%	REP	M	8.3	8.5
		SD	3.6	4.0
7%	REP	M	8.6	8.8
		SD	3.6	4.0
8%	REP	M	8.9	9.1
		SD	3.7	4.1
9%	REP	M	9.2	9.4
		SD	3.8	4.2
10%	REP	M	9.5	9.7
		SD	3.9	4.4

*REP, number of repetitions*.

**Table 5:** Number of repetitions for different criteria of a “valid” repetition in men.

**Tolerated decrease in force**			**Tolerated decrease in pulling time**				
			**0–1 s**	**1–2 s**	**2–3 s**	**3–4 s**	**4–7 s**
0%	REP	M	5.4	5.4	5.7	5.7	5.7
		SD	4.8	4.8	5.4	5.4	5.4
1%	REP	M	6.2	6.3	6.5	6.5	6.5
		SD	4.8	4.8	5.3	5.3	5.4
2%	REP	M	6.8	6.9	7.1	7.1	7.1
		SD	4.8	4.8	5.3	5.3	5.4
3%	REP	M	7.7	7.7	8.0	8.0	8.0
		SD	4.4	4.4	4.9	4.9	5.0
4%	REP	M	8.3	8.3	8.5	8.5	8.6
		SD	4.2	4.2	4.7	4.7	4.8
5%	REP	M	9.2	9.3	9.5	9.5	9.5
		SD	4.1	4.0	4.6	4.6	4.6
6%	REP	M	9.8	9.9	10.1	10.1	10.2
		SD	3.9	3.9	4.4	4.4	4.4
7%	REP	M	10.1	10.2	10.5	10.5	10.6
		SD	3.9	3.9	4.4	4.3	4.3
8%	REP	M	10.5	10.6	10.9	10.9	11.0
		SD	3.8	3.9	4.4	4.3	4.3
9%	REP	M	10.6	10.8	11.1	11.1	11.2
		SD	3.9	3.9	4.5	4.3	4.3
10%	REP	M	10.7	10.9	11.2	11.3	11.4
		SD	3.9	3.9	4.5	4.4	4.3

*REP, number of repetitions*.

In men, the repetition numbers increased even further when more than 3 s of time deviation was tolerated. This means that in some attempts they have just met half of the specified time period of 7 s. As in the women, almost twice as many repetitions were valid in the men when tolerating 10% force deviation than without any tolerance. However, the men already achieved almost the same value at 5% tolerated deviation as at 10% tolerated deviation.

### Correlation Between Lead Climbing Ability and Number of Repetitions

In Table 6 the results of the correlations between the lead climbing ability and the number of repetitions are shown for women and for men. Since the number of repetitions hardly differed between the tolerated time deviation of 3 and more seconds, the results for all force conditions and for time deviations up to 3 s are presented.

**Table 6:** Results of the correlations of the intermittent finger flexor muscle endurance test with lead climbing performance for different tolerated deviations of time and force.

**Condition**		**Women**		**Men**	
**Time Deviation**	**Force deviation**	**R**	**P**	**R**	**P**
0–1 s		0.418^*^	0.030	0.681^*^	<0.001
1–2 s	10%	0.369	0.058	0.678^*^	<0.001
2–3 s		0.371	0.056	0.661^*^	<0.001
0–1 s		0.429*	0.026	0.684^*^	<0.001
1–2 s	9%	0.378	0.052	0.671^*^	<0.001
2–3 s		0.380	0.051	0.647^*^	<0.001
0–1 s		0.425^*^	0.027	0.677^*^	<0.001
1–2 s	8%	0.381	0.050	0.667^*^	<0.001
2–3 s		0.383	0.049	0.640^*^	0.001
0–1 s		0.389^*^	0.045	0.679^*^	<0.001
1–2 s	7%	0.354	0.070	0.668^*^	<0.001
2–3 s		0.354	0.070	0.627^*^	0.001
0–1 s		0.341	0.081	0.691*	<0.001
1–2 s	6%	0.307	0.119	0.682^*^	<0.001
2–3 s		0.307	0.119	0.640^*^	0.001
0–1 s		0.289	0.144	0.669^*^	<0.001
1–2 s	5%	0.260	0.190	0.661^*^	<0.001
2–3 s		0.260	0.190	0.620^*^	0.001
0–1 s		0.261	0.189	0.626^*^	0.001
1–2 s	4%	0.235	0.239	0.619^*^	0.001
2–3 s		0.235	0.239	0.584^*^	0.002
0–1 s		0.157	0.435	0.638^*^	0.001
1–2 s	3%	0.134	0.505	0.631^*^	0.001
2–3 s		0.134	0.505	0.595^*^	0.002
0–1 s		0.045	0.823	0.632^*^	0.001
1–2 s	2%	0.030	0.881	0.626^*^	0.001
2–3 s		0.030	0.881	0.593^*^	0.002
0–1 s		0.066	0.745	0.634^*^	0.001
1–2 s	1%	0.063	0.753	0.631^*^	0.001
2–3 s		0.063	0.753	0.600^*^	0.002
0–1 s		0.033	0.871	0.549^*^	0.005
1–2 s	0%	0.030	0.880	0.549^*^	0.005
2–3 s		0.030	0.880	0.523^*^	0.009

* *Significant, bold = highest correlation for women and men, respectively*.

Obviously, irrespective of the tolerated deviation for the pulling time, the correlation became higher the more force deviation was tolerated. However, a ceiling effect was found at about 8% force deviation for women and about 5% force deviation for men, respectively. Further, for the men no matter which criterion one used, the correlation with lead climbing performance was always above 0.50. For the women, it was always below 0.50.

For the women, test performance did not correlate significantly with climbing performance unless at least a 7% force deviation was tolerated. The highest correlation, *R* = *0.429*, was found for the condition of a tolerance of the pulling time of up to 1 s with a tolerated force deviation of 9%. According to Cohen (1988), a small correlation is found in the women from a force deviation of 3%, and a medium correlation from 6%. For the men, the choice of force deviation tolerance was not quite so decisive. Except for the 0% condition all correlations were higher than 0.6 and thus represented a large correlation. As with the women, the strictest criterion for the pulling time improved their respective correlation. The highest correlation, *R* = *0.691*, was found for the condition of a tolerated decrease in pulling time of 1 swith a decrease of force of 6%.

### Predictability of Lead Climbing Performance by Maximum Finger Strength and Endurance

As shown, in men, the correlation between the number of repetitions in the endurance test and lead climbing performance is much higher than in women. This means that endurance is clearly more relevant for men than for women concerning lead climbing performance. In men, lead climbing performance is predicted by almost 70%, whereas in women it is predicted by no more than 50%. The regression analyses revealed that lead performance is additionally strongly influenced by relative maximum strength in women, but not in men. For women, the *R*^2^ for the overall model was 0.315 (*adjusted R*^2^ = 0.255). The beta coefficient of endurance at 9% tolerated force deviation and 1 s deviation in pulling time was 0.388 (*P* = 0.035), while the beta coefficient of maximum strength was 0.414 (*P* = 0.025). The explained lead climbing performance for men was higher than for women, *R*^2^ = 0.463 (*adjusted R*^2^ = 0.407). However, the beta coefficient of endurance at 6% tolerated force deviation and 1 s deviation in pulling time was 0.682 (*P* < 0.001), whereas the beta coefficient of maximum strength was −0.003 (*P* = 0.988).

## Discussion

Based on the retrospective analysis, the intermittent finger flexor muscle endurance test could be optimized with regard to its validity for the correlation between finger endurance and lead climbing performance. While there has been great evidence to date that continuous test results correlate strongly with climbing performance, as far to our knowledge, no other study than ours has found significant correlations between the number of repetitions from intermittent tests and climbing performance. This might be due to unoptimized previous intermittent test protocols or participants of higher ability level in some previous studies. In the study with male elite and higher elite climbers by Rokowski et al. (2021), much lower, non-significant correlations were found between the time within the force target zone or the force-time integral and onsight and redpoint climbing performance in an intermittent test. In the study by Baláš et al. (2021) that also used an intermittent test and presented the relationship with lead climbing ability, a very similar correlation value as ours (R = 0.656) was obtained for a similar climbing level of the male participants, but only for the force-time integral. They did not consider the number of repetitions. There, the test was performed with a contraction relief ratio of 8:2 s at 60% MVC. The criterion for the termination of the test was, when the force dropped by more than 10% below the target force for more than 1 s, which is pretty much in line with the recommendations we derive from our results. Comparative results for female participants are not yet available.

In the studies on sustained contractions listed above, correlations are sometimes higher (Baláš et al., 2012; López-Rivera and González-Badillo, 2012), sometimes similar (Bergua et al., 2018; Michailov et al., 2018; Baláš et al., 2021) and sometimes lower (Ozimek et al., 2016, 2017). A meaningful comparison appears difficult, as the climbing levels of the test participants vary greatly. However, one can be quite content if a single test already explains 50–70% of the variance in climbing ability.

As in other studies, we considered maximum finger strength as a factor in lead climbing ability in addition to endurance. The regression analyses showed quite different results for men and women. Maximum strength was evidently more decisive (β = 0.357) for women than for men, for whom it is not a determining factor for lead performance at this climbing level (β = −0.001). This seems to contrast with some other studies that attribute a significant portion of climbing performance to maximum finger strength (e.g., Ozimek et al., 2017; Saul et al., 2019). However, the fact that maximum strength is more important for women than for men is also supported by Baláš et al. (2012), in which, for women, relative grip strength explained more than 50% of lead climbing performance but <30% for men.

Based on our results, we can make the following recommendations for intermittent finger flexor muscle endurance testing when using 60% MVC and 7:2 s contraction-rest ratio: For women, we propose to tolerate at least 7% up to 10% force deviation in relation to the predetermined force (significant moderate correlations). Since women pull fewer kilograms in absolute terms, the 5% tolerance is quickly undercut. Otherwise, the test measures not so much endurance as the ability to target the prescribed load very precisely. For men, on the other hand, a smaller percentage force deviation, e.g., of 5%, could also be chosen for test termination. The highest correlation value was reached for 6%, but all considered deviations showed strong correlations. Therefore, the threshold could be set to 10% for both genders for user friendliness.

In any case, it is necessary to be able to provide direct visual feedback on the force-time progression. Already 15 years ago, MacLeod et al. (2007) used “traffic lights” for feedback to maintain the correct force, showing green for correct force, blue for excessive force, and red for too little force. Nowadays, this is realized by means of a display that shows the force-time curve and the threshold line and can be monitored by the athlete, as is the case with our measuring system as well as with others (e.g., Michailov et al., 2018). For the practical test procedure, it is disadvantageous to have the participants perform many more repetitions than finally counted as valid after the retrospective analysis. Optimally, therefore, for example an acoustic or optic signal should be given, when the criteria for a valid repetition are no longer met as in Michailov et al. (2018).

With regard to the temporal extent of the force application, it should be noted that the specified pulling time was hardly ever completely maintained, because at the start of the test the force must be built up. At the end of the test, when experiencing fatigue, differences between men and women occurred. While the women performed almost no repetitions with more than 1 s time deviation, men performed more improper repetitions. Since a realized pulling time that was up to 1 s less than the specified time yielded the highest correlations with lead climbing performance, the consequence from our results is that the test should be terminated rather quickly after the applied force falls below the threshold. A tolerance of 1 s dropping below the threshold as in MacLeod et al. (2007) or Baláš et al. (2021) seems adequate. A two-second wait, as realized in some studies (e.g., Philippe et al., 2012), leads to a reduced correlation with lead climbing performance, especially in men, according to our results.

Unlike continuous tests, which assess local muscle anaerobic capacity, intermittent tests, like the one we used, can be considered a functional measure of climbers' local muscle aerobic capacity. One study suggests that local aerobic capacity is less important than local anerobic capacity in elite climbers. However, in accordance with Fryer et al. (2015c) and Fryer et al. (2016) our study suggests that local aerobic capacity is important for climbers at lower ability level.

Summing up, we think that the application of our recommendations can increase the intermittent tests' validity with respect to climbing performance and climbing specific endurance.

### Limitations of the Study

A limitation of the study is that in the intermittent test, participants could choose whether or not to shake their arms during the 2-s rest after each load phase, as this compromises the standard conditions. However, there were hardly any cases in which the participants did not put their arms down and shake them briefly, so we assume that the influence on the results is not too great.

We did not tell the participants in our study that we were applying different criteria retrospectively as to when a repetition was valid. They assumed that they had to pull the given force over the 7 s. Of course, we could do it the other way around and give participants different target zones to stay within. However, we think that in practice it is quite difficult to target an exact force value and therefore it makes sense to prescribe an exact value but to tolerate a certain deviation. However, further studies could investigate the effects of prescribing different target zones.

Further research could also be conducted to further increase the intermittent test's validity. For example, we did not analyze different combinations of contraction and relaxation phases (e.g., 8:2, 7:3, 7:2 s, etc.). Also, we focused only on the final phase for test termination. One could also consider the initial phase of the test as a criterion for test termination.

The only dependent variable we considered in our study was the number of repetitions, rather than time till task failure and force-time integral, as in many studies cited above. This is because our approach of calculating a valid repetition retrospectively makes these values constants rather than variables. Once the termination criteria are fixed, these two parameters should also be used as performance criteria.

A relatively large number of participants took part in the study. Fortunately, many female participants, who are underrepresented in many studies, could also be recruited. However, the external validity of the findings can only refer to athletes of the climbing levels included in the study, in our case mainly advanced and intermediate athletes. Certainly, especially at the elite level, findings on the validity of performance tests would also be highly desirable.

## Data Availability Statement

The original contributions presented in the study are included in the article/supplementary materials, further inquiries can be directed to the corresponding author.

## Ethics Statement

The studies involving human participants were reviewed and approved by University Augsburg, Ethics Committee. The patients/participants provided their written informed consent to participate in this study.

## Author Contributions

CA, MW, and SK: conception and preparation of the manuscript. MW and CA: performance of work, interpretation, and analysis of data. CA: supervision. All authors contributed to the article and approved the submitted version.

## Funding

This work was supported by the German Federal Institute for Sport Science under Grant ZMVI4-070705/20-21.

## Conflict of Interest

The authors declare that the research was conducted in the absence of any commercial or financial relationships that could be construed as a potential conflict of interest.

## Publisher's Note

All claims expressed in this article are solely those of the authors and do not necessarily represent those of their affiliated organizations, or those of the publisher, the editors and the reviewers. Any product that may be evaluated in this article, or claim that may be made by its manufacturer, is not guaranteed or endorsed by the publisher.
